# Development of an Emergency Locking Unit for a Belt-In-Seat (BIS) System Using a MEMS Acceleration Sensor

**DOI:** 10.3390/s100403759

**Published:** 2010-04-14

**Authors:** Chang Hyun Baek, Jeong Wan Lee, Seock Hyun Kim, Insu Paek

**Affiliations:** Department of Mechanical and Mechatronics Engineering, Kangwon National University, 192-1, Hyoja2-dong, Chuncheon, Kangwon-do, 200-701, Korea; E-Mails: bch2000@kangwon.ac.kr (C.H.B.); jwlee@kangwon.ac.kr (J.W.L.); seock@kangwon.ac.kr (S.H.K.)

**Keywords:** belt in seat, retractor, emergency locking mechanism, MEMS acceleration sensor

## Abstract

This paper proposes an emergency locking unit (ELU) for a seat belt retractor which is mounted on the back frame of a vehicle seat. The proposed unit uses a recliner sensor based on a MEMS acceleration sensor and solenoid mechanism. The seat has an upper frame supported to tilt on a lower frame. The retractor in belt in seat (BIS) system is supported by the upper frame. The proposed recliner sensor based on a MEMS acceleration sensor comprises orientation means for maintaining a predetermined orientation of emergency relative to the lower frame independently of the force of gravity when the upper frame tilts on the lower frame. Experimental results show that the developed recliner sensor unit operates effectively with respect to rollover angles. Thus, the developed unit will have a considerable potential to offer a new design concept in BIS system.

## 1. Introduction

Seatbelts are the most effective means of saving lives by reducing the serious injuries in car accidents. Wearing them properly is also the law in most countries. Commonly used seatbelts are fitted in a B-pillar and are locked by a buckle. Current research, however, confirms that the typical B-pillar type of seatbelt generates low seatbelt wearing rates in some cases [1,2]. One of the major reasons for this is the ‘racking down’ phenomenon occurring in severe cases [3–5]. Further distraction occurs as a result of the seatbelt rubbing or chaffing across the driver's neck and shoulder [6–8].

Seats fitted with Belt-In-Seat (BIS) configuration provide the safest seatbelt combination and ensure that the sash-shoulder rubbing problem is largely eliminated. Also, the problem of retractor locking is greatly reduced because the temporary locking of the retractor does not occur to the same extent [9]. Discomfort issues can therefore be significantly reduced by installing seatbelts on the seat itself. This system is allegedly safer in the case of rollover, especially with four- to eight-year-old children. In terms of design, the BIS system can easily be applied to various models, and it has a more fancy exterior design than the general B-pillar type of seatbelt. Recently, most cars use Center of Gravity (CG) type emergency lock to tighten the seatbelt when the body of the car inclines to a degree over a permissible one.

In a general seatbelt system, the CG type emergency lock is installed in the B-pillar seatbelt to measure the permissible degree of the car’s incline in the design type of a four-bar linkage. However, it is impossible to apply this type of CG type emergency lock to the BIS system because the CG type emergency lock installed in BIS system estimates not the angle of the car body but the angle of recliner. Therefore, a new CG type emergency lock design is required to estimate the absolute degree of incline for the BIS system.

In this paper, we will present our experience on the use of a new design of the emergency locking unit for a BIS system. We designed a recliner sensor which estimates the absolute degree of incline of the car body based on an MEMS accelerometer.

## 2. CG Type Emergency Lock in the BIS System

### 2.1. Description of Conventional Seat Belt System and BIS System

**Figure 1.**
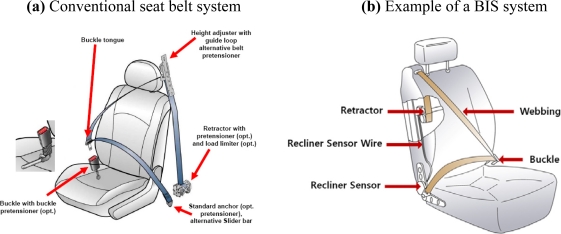
Conventional seat belt system and BIS system [10,11].

Current conventional seat belt systems are three-point belts, in which the upper anchor of the shoulder belt is mounted on the vehicle body (B-pillar), see Figure 1(a).

Properly fastened seat belts distribute the forces of rapid deceleration over larger and stronger parts of the person’s body such as chest, hips and shoulders. The seat belt stretches slightly to slow the body down and to increase its stopping distance. The location of the belt at the occupant’s body during a crash is essential for proper functioning of the belt. For example, if the lap belt is located too high, the occupant can slip under a loosely tightened seat belt, which is called a ‘racking down’ or ‘sub-marining’ effect [6]. More and more belt systems are integrated in the seats and multi-functional, e.g., work in all different types of accidents such as frontal, side, rear end collisions and rollovers.

A BIS system has the upper anchor of the shoulder belt mounted on the top of the seat back frame, see Figure 1(b). BIS systems provide better belt fit, better belt access and greater comfort to the occupants and therefore add to customer satisfaction. A seat belt retractor may be incorporated in a seat back.

Design targets for BIS systems for optimal occupant protection were among others studied by Ford Motor Company [12]. Also in case of a BIS system, the stiffness of the seat and the floor underneath the seat plays a significant role in protection for frontal impact situations. The operation of a prototype BIS system, equipped with a belt pretensioner, load limiting retractor and additional dual stage driver airbags, was investigated by using computer simulations with the simulation package MADYMO [12]. Seat excursion, referred to as the total forward displacement of the shoulder belt upper anchor relative to the vehicle, was shown to be an important parameter to optimize the BIS system and should be limited. Proper structural design of the seat, seat attachment and structural design of the floor were indicated as the key parameters to influence seat excursion. When choosing seat excursion as a design parameter, it is important to make a distinction between the contribution to excursion of the seat and its underlying structure. Since seat excursion is also dependent on vehicle pulse and pitch, the design targets of seat/floor stiffness cannot be generic for all types of vehicles. Proper selection of belt retractor, airbag vent size and dual stage inflator’s lag time contribute to lower injury values.

### 2.2. CG Type Emergency Lock in Conventional Seat Belt Systems

**Figure 2.**
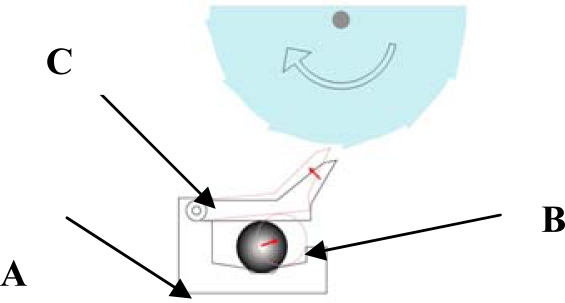
Schematic of a CG type emergency lock.

In recent seat belt systems, a CG type emergency lock is installed in the combination of retractor as a part of seat belt. The basic function of a CG type emergency lock is to tighten the belt when the car body inclines over a permissible angle (12−27°). A typical CG type lock unit consists of three main parts (Figure 2): a ball cup (A) is attached at the B-pillar for adjusting the relative static angle of the car body, a steel ball (B) is in the ball cup to adjust the absolute angle of gravity, and the pawl mechanism locks (C) the belt when the ball slides over the hall. These three parts are combined to form a complete CG type emergency lock unit.

**Figure 3.**
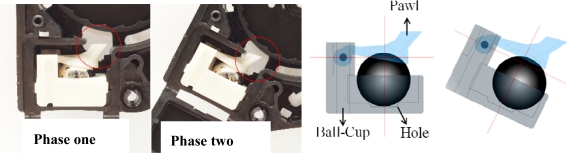
CG type emergency lock schematic motion.

The complete CG type emergency lock is attached to the B-pillar by the retractor unit. The motion of a CG type emergency lock unit is shown schematically in Figure 3. During the first phase, as shown in Figure 3, the upper pawl of the CG type emergency lock does not move, thereby the belt moves freely. Whereas during the second phase, the upper pawl does move, thereby the belt is locked by the force of the gear tooth in the retractor unit. When the car is in the state of rollover accident, the CG type emergency lock goes to the second phase depending on the permissible angle. To be sure, the CG type emergency lock is in the first phase when the car is in a stable state.

### 2.3. Recliner Sensor of BIS Systems

**Figure 4.**
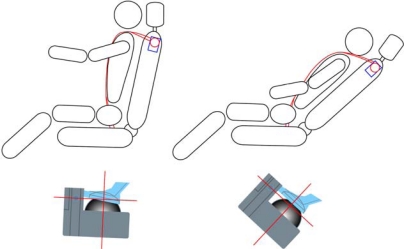
Operation of CG type emergency lock in BIS system.

In a case that the seat belt retractor is incorporated in the seat back, the CG type emergency lock cannot be employed. That is, when the seat back is inclined, the CG type emergency lock is also inclined, so that the locking mechanism may be actuated. As shown in Figure 4, the belt is tightened when the car body does not incline over the permissible degree but the back frame of seat inclines over the permissible one. The recliner sensor unit is controlled by the unwinding or winding of the cable so that the CG emergency locking unit in retractor always stands perpendicular.

## 3. A New Design of an Emergency Locking Unit in a BIS System

### 3.1. Theoretical Background

The reason why MMA7260 was chosen was that although among MEMS acceleration sensors its performance is not excellent, it is a low-cost product and considered suitable for a mass production to substitute the CG type emergency lock.

**Figure 5.**
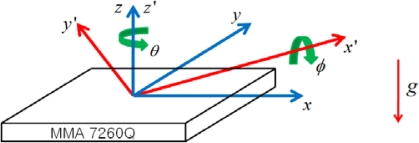
Rotation and force of an acceleration sensor.

Figure 5 shows directions of basic forces that will act on the acceleration sensor in reality. It describes a situation where a car is rotating by an angle of *θ* about z-axis (or z’-axis), and by an angle of *φ* about x’-axis which is the x-axis rotated about z-axis by *θ*. In the figure, *φ* represents an incline of the car (The car is assumed to face positive x direction). The acceleration and inclination of the car can be estimated using:

(3.1) $$\begin{array}{l} a_{x}=g\;\text{sin}\;\phi \;\text{cos}\;\theta \\ a_{y}=-g\;\text{sin}\;\phi \;\text{sin}\;\theta \end{array}$$

Equation (3.1) can be rewritten as

(3.2) $$a_{x}^{2}+a_{y}^{2}=g^{2}\;{\text{sin}}^{2}\;\phi$$

If a target angle, *ψ*, is introduced to Equation (3.2), the equation for a control unit operation can be expressed as follows:

(3.3) $$a_{x}^{2}+a_{y}^{2}=g^{2}\;{\text{sin}}^{2}\;\phi \geq g^{2}\;{\text{sin}}^{2}\;\psi$$

If Equation (3.3) is satisfied, the emergency locking unit (ELU) should start operating.

**Figure 6.**
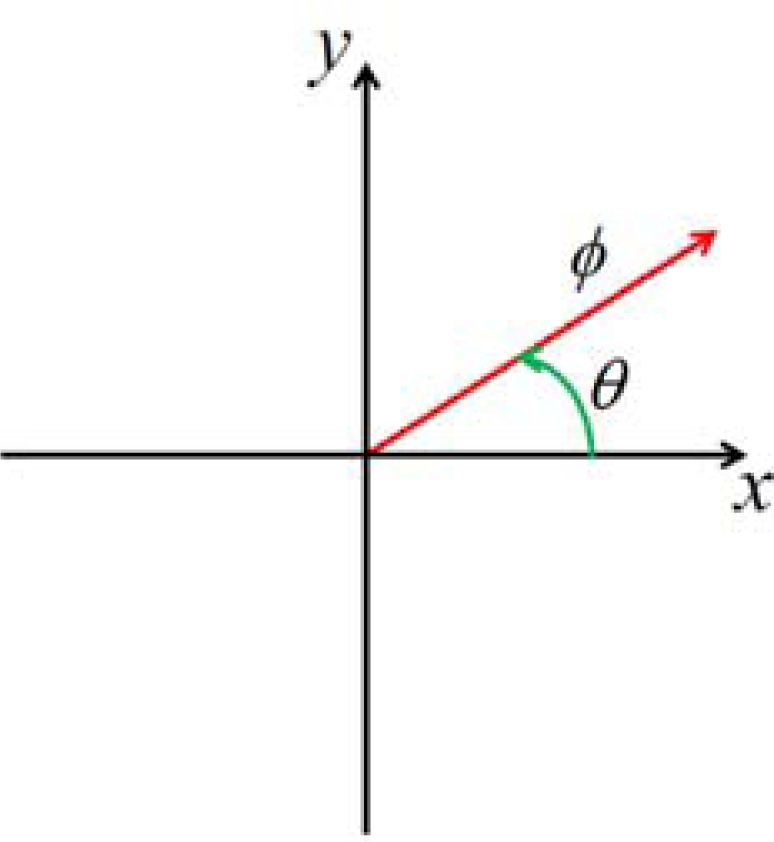
Relation between x-y coordinates and angles of *ϕ* and *θ*.

Figure 6 shows a relationship between x-y coordinates and angles of *ϕ* and *θ* to evaluate Equation (3.3), which is represented by:

(3.4) x=ϕ cos θ,  y=ϕ sin θ

**Figure 7.**
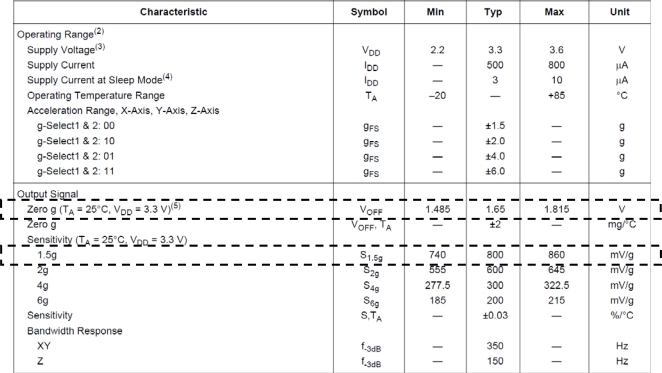
MMA7260Q’s Datasheet (Freescale Co.).

In reality, data from acceleration sensors cannot be represented by a simple equation such as Equation (3.3) due to an offset and sensitivity. Figure 7 shows a datasheet of the acceleration sensor (MMA7260, Freescale Co.) used in the study. The data in the dashed boxes of the figure are the offset and sensitivity of the sensor. Although the offset and sensitivity of the sensor depend on the temperature, their temperature dependences are very small and ignored in estimating acceleration from the measured signal.

Including an offset and sensitivity of the sensor, the acceleration from the sensor can be estimated from [13,14]:

(3.5) $$\begin{array}{l} a_{x}=g\beta_{x}\;\text{sin}\left(\phi +\alpha_{x}\right)\text{cos}\;\theta \\ a_{y}=-g\beta_{y}\;\text{sin}\left(\phi +\alpha_{y}\right)\text{sin}\;\;\theta \end{array}$$

where, a is the acceleration, α is the offset, and β is the sensitivity. Subscripts x and y represents x and y directions, respectively. From Equations (3.3) to (3.5), the equation for a control unit operation can be rewritten as:

(3.6) $$\frac{{\left(x+\frac{\alpha_{x}}{\sqrt{x^{2}+y^{2}}}\;x\right)}^{2}}{{\left(\frac{\psi }{\beta_{x}}\right)}^{2}}+\frac{{\left(y+\frac{\alpha_{y}}{\sqrt{x^{2}+y^{2}}}\;y\right)}^{2}}{{\left(\frac{\psi }{\beta_{y}}\right)}^{2}}\;\geq 1$$

Finally, the ELU will start operating in situations where Equation (3.5) is satisfied.

### 3.2. Overall System

**Figure 8.**
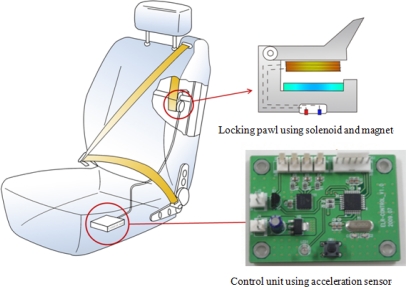
Overall system developed for this study.

Instead of using a mechanical recliner sensor such as TAKADA’s system, a control unit with a MEMS acceleration sensor (Freescale Co., Model MMA7260Q) was developed and used to measure the inclination of the car body. Figure 8 shows the overall system consisting of the new control unit with the acceleration sensor, and a locking pawl with a solenoid and magnet developed for this study. For the developed system, the control unit with the acceleration sensor is installed on the bottom frame of seat, the belt is not tightened although the back frame of seat is inclined.

**Figure 9.**
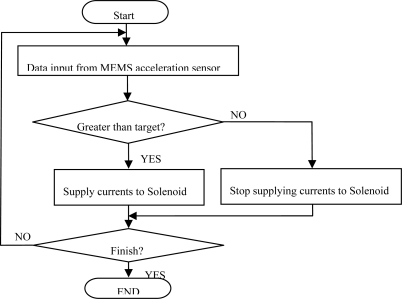
Operation algorithm of the developed system.

Figure 9 shows the flowchart of the control algorithm applied to the developed control unit. The basic principle of this system is as follows: first of all, the acceleration sensor measures the inclination of the car. If the measured value is higher than a certain criterion, currents are supplied to the solenoid, and finally the locking system operates. After tightening the seat belt, the sensor again measures the inclination of the car. If the measured value is lower than the criterion, the system releases the belt by stopping supplying currents to the solenoid.

### 3.3. Experimental Results

Tests including an incline test and acceleration test were performed to find out how the developed recliner sensor unit based on a MEMS acceleration sensor performs. The tests were done based on the regulation in the Korean Seat Belts Standard (KS R 4027). They were intended to simulate any circumstances causing inclination of the car seats such as rollover, running on sharply inclined roads, turning along curved roads, *etc*.

#### 3.3.1. Incline Test

**Figure 10.**
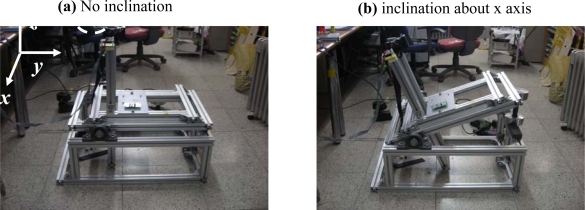
Equipment for incline test.

Figure 10 is a test rig developed for the incline test. Using the test rig, it was tested if the developed system satisfies the inclination regulation in the Korean Seat Belts Standard (KS R 4027). The Real Time Windows Target (V. 2.7) in Matlab Simulink was used for the test. A total of four control units having the same sensors were developed and tested.

For the test, the control unit was installed on top of the vertical frame, and the locking status of the ELU was checked by the voltage reading from the solenoid while rotating the upper part of the test rig x axis. The whole test was repeated while rotating the control unit about z axis from 0° to 337.5° with an interval of 22.5°.

**Figure 11.**
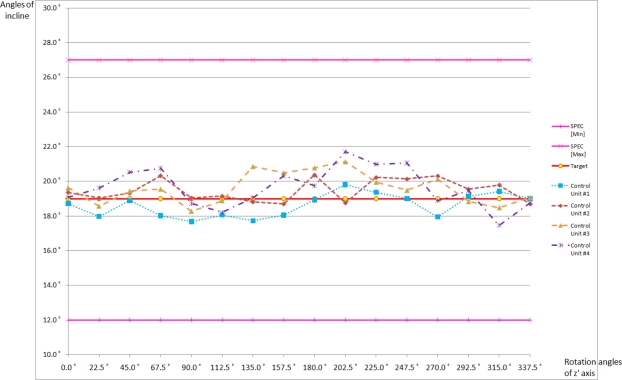
Experiment result of overall control unit.

The target inclination angle for the locking system to operate was set to be 19° for all four control units. Figure 11 shows the inclination angles where the locking systems of the four control units operated as well as minimum (12°) and maximum (27°) values in the regulation. As can be seen in the figure, all four units tested operated within the range specified in the regulation.

The maximum error was 2.7(−) and occurred from control unit 4. The average values of the errors were within +/− 1° for all the units. The main reason to cause different errors for different control units is believed to be due to the uncertainties of the MEMS acceleration sensors.

#### 3.3.2. Acceleration Test

**Figure 12.**
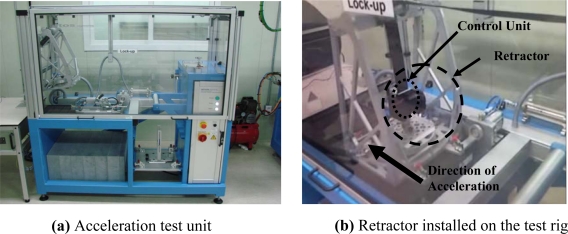
Equipment for acceleration experiment.

The acceleration test was performed using a seat belt acceleration test unit (ATOS Engineering, Model LU-300/24S) having a range of acceleration from 0.1 m/s^2^ (0.01g) to 50 m/s^2^ (5 g). The position resolution and control & sample frequency of the unit are 1 μm/±10 g and 10 kHz, respectively. The picture of the acceleration test unit is shown in Figure 12.

**Figure 13.**
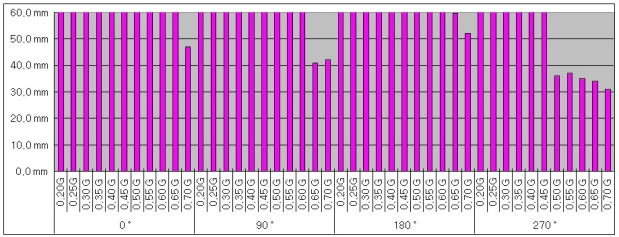
Measured extension of webbing vs. acceleration.

For the test, the control unit and retractor were installed on the test rig as shown in Figure 12(b), and accelerated in y direction. The correct operation of the emergency locking system was checked by the voltage reading from the solenoid at an acceleration of 4.5 m/s^2^ (0.45 g), and the extension of webbing was recorded right after locking. The acceleration from 2 m/s^2^ (0.2 g) to 7 m/s^2^ (0.7 g) with an interval of 0.5 m/s^2^ (0.05 g) was applied. The measurement at each acceleration amplitude was repeated once. Also in the test, the control unit was rotated from 0° to 270° with an interval of 90° about z axis shown in Figure 10, and the whole acceleration test explained beforehand was repeated. One control unit out of four was tested. The test result is shown in Figure 13.

In Figure 13, the x axis represents an acceleration amplitude and direction with a range of 2 m/s^2^ (0.2 g)−7 m/s^2^ (0.7 g) in four directions, and y axis represents the extension of webbing. In order to satisfy the regulation in the Korean Seat Belts Standard (KS R 4027) in the acceleration test, the locking system must operate before the webbing extends 50 mm at 4.5 m/s^2^ (0.45 g). As shown in the figure, however, the locking system started operating at an acceleration greater than or equal to 0.5 g.

The reason why the control unit didn’t work correctly is believed to be due to the fact that the operation algorithm of the control unit didn’t operate properly to supply currents to the solenoid when the acceleration reached 4.5 m/s. This part needs to be revised in the future.

## 4. Concluding Remarks

In this paper, an emergency locking mechanism for a BIS system was developed. The new emergency locking unit is based on a MEMS acceleration sensor and solenoid mechanism. The new recliner sensor unit was designed to be activated in case of a rollover, and to operate primarily relative not to the angle of a back frame of seat but to the one of the body of car. Experimental results show that the developed recliner sensor unit operates effectively with respect to rollover angles. In addition, it reduces mechanical machining in the process of production. Thus, the developed unit is believed to have a considerable potential to offer a new design concept of a BIS system.

## References

[1] Autoliv (2002). Autoliv seat belts fitted to new Renault Megane. AutoTechnology.

[2] Haland Y. (2002). The evolution of the three point seat belt from yesterday to tomorrow. Autoliv Research.

[3] Hassan A., Morris A., Welsh R. Some Characteristics of side impact crashes involving modern passenger vehicles. http://www.ukccis.com/downloads/download_publication.asp?file=publications/ICrash_200621406-rw2.PDF.

[4] Farmer C.M., Wells J.K., Werner J.V. Relationship of head restraint positioning to driver neck injury in rear-end crashes.

[5] Welcher J.B., Szabo T.J. The relationship between seat properties and human subject kinematics in rear impact tests.

[6] Song D., Uriot J., Trosseille P., Mack C., Tarriere C., Got C., Domont A. Modeling and analysis of interactions between occupant, seatback and headrest in rear impact.

[7] Balci R., Vertiz A. (2001). Delphi automotive systems, comfort and usability of the seat belts.

[8] Borde P. (2001). Pyrotechnic knee bolster development and its contribution to car drivers safety.

[9] Viano D.C. (2003). Energy transfer to an occupant in rear crashes: effect of stiff and yielding seats.

[10] *Autoliv* Home Page. http://www.autoliv.com.

[11] *Delphi* Home Page. http://www.delphi.com.

[12] Zhou R., Hong W., Lakshminarayan V. (2001). Design targets for seat integrated restraint systems for optimal occupant protection.

[13] Park S., Jeon M. (2003). A study on improvement of crash discrimination performance for offset and angular crash events using electronic X-Y 2-axis accelerometer. Trans. Korean Soc. Autom. Eng.

[14] Song C.M., Lee J.W. Autocalibration method of three-axis micromachined accelerometers.

